# Current treatment practices and efficacy in solar urticaria: insights from a patient survey

**DOI:** 10.3389/fimmu.2025.1683524

**Published:** 2025-11-04

**Authors:** Lea Kiefer, Felix Aulenbacher, Dorothea Terhorst-Molawi, Ana M. Giménez-Arnau, Margarida Gonçalo, Atsushi Fukunaga, Emek Kocatürk Göncü, Kanokvalai Kulthanan, Sheila McSweeney, Heike Röckmann, Karsten Weller, Lesley E. Rhodes, Manuel P. Pereira

**Affiliations:** ^1^ Institute of Allergology, Charité – Universitätsmedizin Berlin, Corporate Member of Freie Universität Berlin and Humboldt-Universität zu Berlin, Berlin, Germany; ^2^ Immunology and Allergology, Fraunhofer Institute for Translational Medicine and Pharmacology (ITMP), Berlin, Germany; ^3^ Department of Dermatology, Hospital del Mar-Institut d’Investigacions Mèdiques Universitat Pompeu Fabra de Barcelona, Barcelona, Spain; ^4^ Department of Dermatology, University Hospital, Coimbra Local Health and Faculty of Medicine, University of Coimbra, Coimbra, Portugal; ^5^ Division of Dermatology, Department of Internal Related, Kobe University Graduate School of Medicine, Kobe, Japan; ^6^ Department of Dermatology, Koç University School of Medicine, Istanbul, Türkiye; ^7^ Department of Dermatology, Faculty of Medicine Siriraj Hospital, Mahidol University, Bangkok, Thailand; ^8^ St. John’s Institute of Dermatology, Guy’s Hospital, London, United Kingdom; ^9^ Department of Dermatology/Allergology, University Medical Centre Utrecht, Utrecht University, Utrecht, Netherlands; ^10^ Faculty of Biology, Medicine and Health, University of Manchester, and Dermatology Centre, Salford Royal Hospital, NCA NHS Foundation Trust, Manchester Academic Health Science Centre, Manchester, United Kingdom

**Keywords:** Solar urticaria, chronic inducible urticaria, treatment, antihistamines, omalizumab

## Abstract

**Background:**

Solar urticaria (SolU) is a rare chronic inducible urticaria and photodermatosis, presenting with wheal/flare formation accompanied by severe itch, following exposure to light in the triggering action spectrum. Therapeutic options remain limited for SolU, and the perspective of patients regarding the efficacy of available treatments remains unknown.

**Methods:**

Patients with SolU, organized in a disease-specific Facebook group, were asked to complete an electronic questionnaire on their condition and therapies performed between May 2023 and April 2024. The certainty of SolU diagnosis was differentiated as i) physician confirmed by clinical presentation, ii) light provocation tests, or iii) patient-reported. Study outcomes included clinical presentation, triggering action spectrum, disease severity, impairment of quality of life, therapies performed, and their efficacy. Logistic regression models were used to study the association between clinical factors and treatment outcomes.

**Results:**

A total of 112 patients (female, n = 94; median age, 42 years) participated in the study. Most patients considered their condition severe or extremely severe (n = 72, 76.6%) with a very/extremely impacted quality of life (n = 82, 86.3%). The majority of patients received non-sedating antihistamines (58.9%, n = 66), leading to worsening, no change, or only slight improvement in most cases (82.2%, n = 53). Omalizumab was given to 28 patients and induced complete control in 32.1% of cases. Treatments with sedating antihistamines, ciclosporin, systemic corticosteroids, phototherapy, and *Polypodium leucotomos* were performed in a residual number of patients and did not lead to a substantial improvement of the symptoms. Antihistamines were more effective in patients with mild disease, whereas omalizumab maintained a positive response across different disease severity levels.

**Conclusion:**

SolU is generally perceived as severe by affected patients, leading to a high impairment of quality of life. Performed therapies, including off-label treatments, are not sufficient to reach complete remission of symptoms in the majority of patients. Effective therapeutics for SolU are urgently needed to achieve better care for this highly burdened patient population.

## Introduction

Solar urticaria (SolU) is a rare chronic inducible urticaria and photodermatosis with transient lesions induced by components of solar radiation, such as UVA, UVB, or visible light, but also by artificial light sources (1, 2). According to estimations, SolU accounts for less than 0.5% of chronic urticaria cases and 0.7% to 3.2% of all photodermatoses (3). People of all skin types can be affected, and it occurs more frequently in women, with peak presentation at approximately 35 years of age (4). Clinically, burning, itching, erythema, and whealing develop within 5–10 minutes after sun exposure in most patients. SolU is associated with a high disease burden, severely impacting the quality of affected patients (5, 6) due to both the symptoms of the disease and avoidance behaviors (4). Challenge tests are performed using narrowband and broadband irradiation sources, with methodology and availability varying between centers. Photoprovocation enables the confirmation of the diagnosis and the evaluation of minimal urticarial doses (MUDs), which additionally inform SolU severity and action spectrum (4).

Current guidelines recommend the use of a non-sedating antihistamine (nsAH) for the treatment of SolU (7). In general, antihistamines are observed to have variable clinical effects, with a proportion of patients being refractory to single doses and a fourfold dose of these drugs (8). The monoclonal IgE antibody, omalizumab, which prevents the binding of IgE antibodies to the mast cell Fc_ϵ_RI receptor, is reported to induce therapeutic relief in a subset of SolU patients (9–11). The efficacy of omalizumab in SolU and other chronic inducible urticarias is further supported by real-world data (12–15). However, in a phase II open-label study including 10 SolU patients refractory to antihistamines, omalizumab (300 mg/4 weeks) failed to demonstrate efficacy and a substantive increase in MUD (16). Other therapeutic approaches include inducing tolerance by repetitive exposure to increasing doses of UV radiation, as in phototherapy hardening (17), or promoting melanization through treatment with afamelanotide (18). Intravenous immunoglobulins have been considered as a treatment option for SolU but have not yet shown sufficient efficacy (19), while a study suggests that *Polypodium leucotomos* (PL), as a natural treatment option, can reduce skin reactions in SolU (20).

This study was designed as an online survey with the aim of assessing disease characteristics and current treatment practices, as well as the patients’ perspective on the efficacy of available treatment options in SolU. This will help to better understand the disease and its impact on patients’ quality of life, which therapies work best in a real-world setting, and ultimately improve the care of patients with SolU.

## Methods

### Study population

This was a survey-based study conducted between May 2023 and April 2024. A survey was distributed via the social media platform Facebook within a SolU patient group, and all patients with SolU, diagnosed i) by a physician clinically, ii) by photoprovocation, or iii) per self-report, were included in this survey study. There was no age restriction to participate in the survey. Participation was voluntary, and informed consent was obtained electronically prior to the initiation of the survey (see complete survey in the **Supplementary Material**). For underage participants, signed informed consent was provided by their legal guardian. This study was approved by the local ethics committee (Charité – Universitätsmedizin Berlin, EA1/167/22). All study procedures were performed according to the Declaration of Helsinki and later revisions.

### Study design and outcomes

The survey consisted of 36 questions and additional optional questions depending on the received treatments. It was available in German, English, and Spanish. SoCi-Survey was used to develop the online survey (https://www.soscisurvey.de). Patients’ responses to the online survey were automatically stored in a secure database. Participants could skip any questions they felt uncomfortable answering, and the survey was designed to take approximately 10–15 minutes to complete. No identifiable personal information was collected, ensuring participant anonymity throughout the process.

The questionnaire enquired about demographics (age and sex), clinical characteristics (time to diagnosis, action spectrum, overall disease severity, and disease severity at its worst), disease burden (overall impact on quality of life and impact at its worst), and performed therapies (sedating antihistamines, nsAH, omalizumab, ciclosporin, systemic corticosteroids, phototherapy, PL, and other treatment modalities) and their efficacy using a 6-point Likert scale (complete control, significant improvement, moderate improvement, slight improvement, no change, and worsening). When patients received more than one dosage of one particular drug, the effect of the higher dose was considered for analysis.

### Statistical analysis

Statistical analysis was performed using R (version 4.3.2), which provides a robust framework for examining demographic and clinical characteristics. Descriptive statistics were used to analyze the survey data. Categorical data, such as demographic characteristics (e.g., sex and age), and responses related to the action spectrum of SolU were summarized as frequencies and percentages. Continuous data, such as age or time to diagnosis, were summarized using median and range. To investigate the relationships between the studied variables, such as disease severity, quality of life, and time to diagnosis, a correlation analysis using Spearman’s rank correlation coefficient and Kendall’s tau was performed. Additionally, we investigated the association between demographic and clinical factors and the probability of a positive treatment response to nsAH and omalizumab using logistic regression models. A successful treatment was considered when patients evaluated their treatment response as achieving “moderate improvement”, “significant improvement”, or “complete control”; treatments leading to a “slight improvement”, “no change”, or “worsening” were deemed unsuccessful. The statistical significance was set at p < 0.05.

## Results

### Demographics

A total of 112 patients completed the online survey. Most respondents were female [female, n = 94 (83.9%); male, n = 12 (10.7%); unknown, n = 6 (5.4%)], and the median age was 42 years (range, 10–68; n = 104; **Table 1**).

**Table 1:** Demographic and clinical characteristics of the study population (n = 112).

Parameter	Percentage (%)*	Cases (n)
Sex		
◼ Female	83.9	94
◼ Male	10.7	12
◼ Unknown	5.4	6
Age (years)		
◼ <18	1.9	2
◼ 18–30	21.2	22
◼ 31–49	51	53
◼ 50–64	23.1	24
◼ ≥65	2.9	3
Time to diagnosis after symptom onset		
◼ 1 year	37.7	20
◼ >1 year	62.3	33
Diagnosis**		
◼ Clinical symptoms	45.5	50
◼ Light provocation	38.2	42
◼ Self-reported	16.4	18
Time to symptoms (min)		
◼ <1	19.2	20
◼ 1–5	44.2	46
◼ 6–10	22.1	23
◼ 11–20	6.7	7
◼ >20	7.7	8
Action spectrum***		
◼ Known	56.30	63
o UVA	68.3	43
o UVB	65.1	41
o Visible light	41.3	26
o Infrared	4.8	3
o Other	7.9	5
Disease severity		
◼ Extremely mild	2.1	2
◼ Mild	4.3	4
◼ Moderate	17	16
◼ Severe	48.9	46
◼ Extremely severe	27.7	26
Impact on quality of life (overall)		
◼ Little	4.2	4
◼ Moderate	9.5	9
◼ Very strong	27.4	26
◼ Extremely strong	58.9	56
Impact on quality of life (at worst)		
◼ Little	0	0
◼ Moderate	6.3	6
◼ Very strong	18.9	18
◼ Extremely strong	74.7	71

*Percentages were calculated based on the number of responses to each item and not based on the total population.

**The diagnosis was i) physician confirmed by clinical presentation, ii) confirmed by light provocation tests, or iii) patient-reported.

***Multiple responses possible.

### Clinical characteristics

A total of 37.7% (n = 20) of patients were diagnosed within the first year after symptom onset, whereas 62.3% (n = 33) received their diagnosis more than 1 year after symptoms began. Most frequently, the diagnosis was made by a physician according to the clinical presentation (45.5%, n = 50). Light provocation testing was performed in 38.2% (n = 42) of cases, while in 18 patients (16.4%), the diagnosis was self-reported without definitive confirmation. Most patients (85.6%, n = 89) reported a time to reaction up to 10 minutes, and 7.7% (n = 8) reported a reaction time over 20 minutes. In total, 56.3% (n = 63) of respondents stated that they knew their action spectrum, with 40 patients indicating that multiple wavelengths triggered their urticaria. Among these, 68.3% (n = 43) reported reacting to UVA, 65.1% (n = 41) to UVB, 41.3% (n = 26) to visible light, 4.8% (n = 3) to infrared, and 7.9% (n = 5) to other not specified light sources (**Table 1**).

### Disease burden

Most patients considered their disease as severe or extremely severe (76.6%, n = 72). In contrast, only two patients reported extremely mild symptoms (2.1%), with the diagnosis in these two cases being confirmed by a physician but not assessed by photoprovocation. Overall, the impact of SolU on quality of life was considered very strong or extremely strong by the majority of patients (86.3%, n = 82), with even higher impact at peak disease severity (**Table 1**). We recorded a significant moderate correlation between the impact on quality of life and perceived symptom severity (r = 0.46, p < 0.001, *Spearman*) and a weak correlation with time to diagnose (r = 0.23, p = 0.043, *Kendall’s tau*).

### Treatment

**Figure 1:**
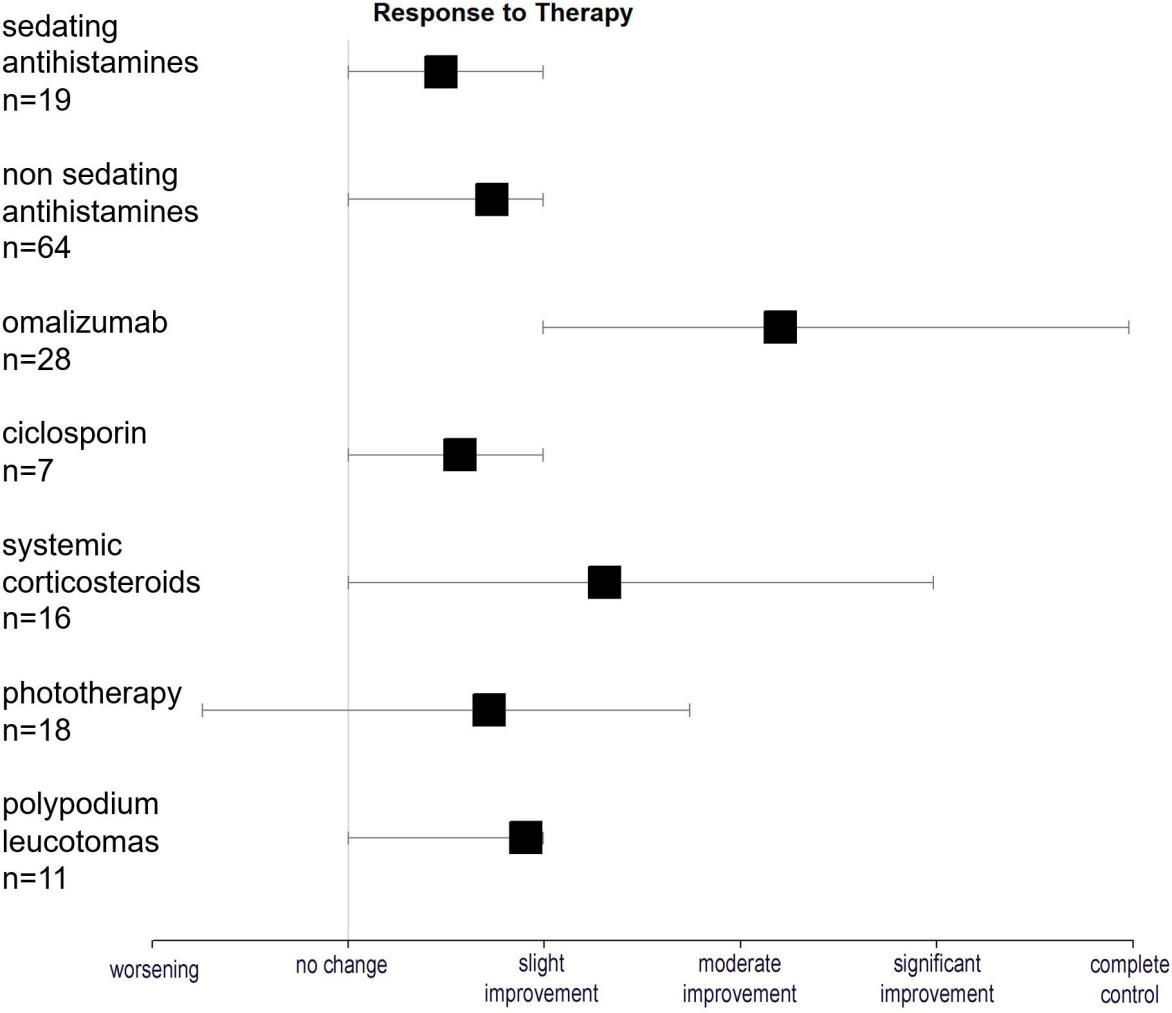
Response to therapy. Forrest plot showing the response to various treatment modalities as assessed by patients (complete control, significant improvement, moderate improvement, slight improvement, no change, and worsening). Black squares represent mean values, and whiskers indicate the 10th and 90th percentiles.

Most patients were treated with nsAH (58.9%, n = 66), with worsening, no improvement, or only slight improvement in most cases (82.2%, n = 53; two missing cases). Omalizumab was given to 25% (n = 28) of patients, with the majority (n = 20) receiving the standard dose of 300 mg/4 weeks. Five patients were treated with an increased dose (>300 mg/4 weeks), while one received 150 mg/4 weeks. Two patients underwent two different drug regimens: 150 mg and 300 mg/4 weeks. Omalizumab induced complete control in 32.1% (n = 9) of cases and significant or moderate improvement in a further 28.6%. Other treatment modalities, including sedating antihistamines, ciclosporin, systemic corticosteroids, phototherapy, and PL, were performed in a residual number of patients and, in general, did not lead to a substantial improvement of symptoms (**Table 2**, **Figure 1**).

**Table 2:** Summary of treatment efficacy.

Treatment modalities	Complete control n (%)*	Significant improvement n (%)*	Moderate improvement n (%)*	Slight improvement n (%)*	No change n (%)*	Worsening n (%)*
Sedating antihistamines	0 (0%)	0 (0%)	1 (5.3%)	**9 (47.4%)**	7 (36.8%)	2 (10.5%)
Non-sedating antihistamines	1 (1.6%)	2 (3.2%)	8 (12.5%)	24 (37.5%)	**26 (40.6%)**	3 (4.7%)
Omalizumab	**9 (32.1%)**	3 (10.7%)	5 (17.9%)	7 (25.0%)	4 (14.3%)	0 (0%)
Ciclosporin	0 (0%)	0 (0%)	1 (14.3%)	2 (28.6%)	**4 (57.1%)**	0 (0%)
Systemic corticosteroids	0 (0%)	**5 (31.3%)**	1 (6.3%)	**5 (31.3%)**	4 (25.0%)	1 (6.3%)
Phototherapy	0 (0%)	2 (11.1%)	3 (16.7%)	**6 (33.3%)**	2 (11.1%)	5 (27.8%)
*Polypodium leucotomos*	0 (0%)	1 (9.1%)	1 (9.1%)	**5 (45.5%)**	4 (36.4%)	0 (0%)

Patients rated the efficacy of various treatment options as leading to complete control, significant improvement, moderate improvement, slight improvement, no change, or worsening. For each treatment modality, the most frequently reported response is highlighted in bold.

*Percentages were calculated based on the number of responses to each item and not based on the total population.

Logistic regressions revealed an association of an unknown action spectrum and a diagnosis based on clinical evaluation (without photoprovocation) with a poor treatment response to nsAH (**Table 3**). As for omalizumab, there was no statistically significant association between clinical and demographic factors and therapy response (**Table 4**). Antihistamines seemed to be more effective in patients with mild disease, as patients with mild disease had a higher probability (72.1%) of having a positive treatment response to nsAH compared to those with a severe condition (3.5%). As for omalizumab, the likelihood of a positive response was maintained across severity levels. Moreover, patients diagnosed solely on clinical symptoms had a lower response probability to nsAH (19.2%), but not to omalizumab.

**Table 3:** Logistic regression analysis evaluating the association between demographic and clinical factors with the treatment efficacy of non-sedating antihistamines.

Variable	Type	Estimate (β)	Standard error	Z-Statistic	p-Value	Estimated probability
Time to diagnosis after symptom onset	Intercept	−1.326	0.742	−1.787	0.074	0.210
	Effect	−0.079	0.222	−0.354	0.723	0.142
Age (years)	Intercept	−0.745	1.013	−0.736	0.462	0.322
	Effect	−0.213	0.272	−0.782	0.434	0.117
Disease severity (overall)	Intercept	0.948	1.273	0.745	0.457	0.721
	Effect	−0.611	0.322	−1.898	0.058	0.035
Disease severity (at worst)	Intercept	−0.854	1.509	−0.566	0.571	0.299
	Effect	−0.135	0.304	−0.444	0.657	0.142
Impact on quality of life (overall)	Intercept	0.291	1.507	0.193	0.847	0.572
	Effect	−0.298	0.251	−1.188	0.235	0.142
Impact on quality of life (at worst)	Intercept	−1.593	2.007	−0.794	0.427	0.169
	Effect	0.012	0.307	0.040	0.968	0.181
Known action spectrum	Intercept (Ref: No)	−1.386	0.559	−2.480	0.013	0.200
	Yes	−0.194	0.696	−0.279	0.780	0.171
Diagnostics	Intercept (Ref: Clinical symptoms)	−1.435	0.498	−2.884	0.004	0.192
	light provocation	−0.174	0.698	−0.250	0.803	0.167
	diagnosis suspected	0.049	1.224	0.040	0.968	0.200

A successful treatment was considered when patients evaluated their treatment response as achieving “moderate improvement”, “significant improvement”, or “complete control”; treatments leading to a “slight improvement”, “no change”, or “worsening” were deemed unsuccessful. A positive beta represents a positive association, while a negative beta represents an inverse association.

Each row represents a logistic regression model assessing the association between one clinical or demographic variable and treatment success. The Intercept indicates the estimated baseline log-odds of treatment success when the variable is at zero (or at its reference level). The Effect row shows how the predictor changes the log-odds. Positive values indicate higher odds of treatment success; negative values indicate reduced odds.

**Table 4:** Logistic regression analysis evaluating the association between demographic and clinical factors with the treatment efficacy of omalizumab.

Variable	Type	Estimate (β)	Standard error	Z-Statistic	p-Value	Estimated probability
Time to diagnosis after symptom onset	Intercept	1.078	1.033	1.043	0.297	0.746
	Effect	−0.226	0.237	−0.953	0.341	0.431
Age (years)	Intercept	3.361	1.605	2.094	0.036	0.966
	Effect	−0.790	0.405	−1.949	0.051	0.201
Disease severity (overall)	Intercept	1.817	1.709	1.063	0.288	0.860
	Effect	−0.310	0.371	−0.836	0.403	0.413
Disease severity (at worst)	Intercept	3.870	2.106	1.837	0.066	0.980
	Effect	−0.673	0.396	−1.699	0.089	0.301
Impact on quality of life (overall)	Intercept	−0.166	2.289	−0.073	0.942	0.459
	Effect	0.086	0.357	0.240	0.811	0.606
Impact on quality of life (at worst)	Intercept	1.195	2.737	0.437	0.662	0.768
	Effect	−0.115	0.410	−0.281	0.779	0.596
Known action spectrum	Intercept (Ref: No)	1.099	1.155	0.951	0.341	0.750
	Yes	−0.762	1.227	−0.621	0.534	0.583
Diagnostics	Intercept (Ref: Clinical symptoms)	0.511	0.730	0.699	0.484	0.625
	light provocation	−0.105	0.861	−0.122	0.900	0.600

A successful treatment was considered when patients evaluated their treatment response as achieving “moderate improvement”, “significant improvement”, or “complete control”; treatments leading to a “slight improvement”, “no change”, or “worsening” were deemed unsuccessful. A positive beta represents a positive association, while a negative beta represents an inverse association.

Each row represents a logistic regression model assessing the association between one clinical or demographic variable and treatment success. The Intercept indicates the estimated baseline log-odds of treatment success when the variable is at zero (or at its reference level). The Effect row shows how the predictor changes the log-odds. Positive values indicate higher odds of treatment success; negative values indicate reduced odds.

## Discussion

Patients with SolU generally perceive their condition as severe, with a substantial impact on quality of life. Most patients are treated with antihistamines, which, however, show an insufficient effect in most cases. Omalizumab seems to be more effective, but it is not approved for the treatment of SolU and, thus, is only accessible for a subset of patients as an off-label therapy.

Most study participants were diagnosed based on clinical symptoms, and only a subset of patients underwent light provocation testing. These findings outline the discrepancy in diagnostic work-up across clinical centers and may reflect the lack of photoprovocation devices in non-specialized centers. As a result, a substantial proportion of patients (44%) reported not knowing their action spectrum, potentially affecting their understanding of their triggering factors and their quality of life. This suggests a need to better standardize the diagnostic work-up in SolU, including the implementation of photoprovocation. Moreover, most patients experienced a diagnostic delay of over 1 year after the onset of symptoms. This delay may result from a lack of awareness of SolU in routine dermatological and allergy clinics, the time taken for general practitioners to refer to a dermatology center, and limitations in access to specialized centers. Furthermore, patients experience their symptoms mostly during spring and summer, so there may be a seasonal delay.

Most SolU patients rated their symptoms as severe and the impact of their disease on their quality of life as very high, consistent with reports of previous observations from photodermatology units (4–6). Our study revealed, as expected, a significant positive correlation between disease severity and the impact on quality of life (r = 0.46, p < 0.001), suggesting an association between these two parameters. Other factors, such as avoidance behaviors with social isolation, visits to doctors’ appointments, and out-of-pocket costs, should also be taken into consideration and assessed, as they may contribute to an impairment in health-related quality of life.

nsAH was the treatment most often offered to patients, showing a broad spectrum of perceived efficacy, with patients reporting both successful and unsuccessful outcomes with these drugs. Omalizumab induced more often complete control of SolU (32.1%) compared to all other treatment modalities. These results support previous studies investigating the effect of omalizumab on different forms of chronic inducible urticaria (especially symptomatic dermographism, cold urticaria, and SolU), in which this biologic drug showed a rapid onset of action with good symptom control in a substantial proportion of patients, but not in all (12–14).

Other treatment modalities were only performed in a small subset of patients, and thus, the results should be regarded with caution. The majority of patients undergoing phototherapy (61.1%, n = 11) experienced a slight to significant improvement of symptoms, while a small number (27.8%, n = 5) reported a worsening of the SolU, which raises concerns about its suitability for some patients. Systemic corticosteroids led to an improvement of the symptoms in most patients, but their long-term use cannot be recommended due to their unfavorable safety profile. The natural treatment alternative PL may be considered in SolU, as it seemed to improve the condition in some patients.

Notably, our findings indicate that therapeutic response to nsAH is associated with disease severity, whereas omalizumab efficacy remains consistent across severity levels, although in a relatively small percentage of patients, suggesting a relevant role of IgE-dependent mechanisms in the pathophysiology of a subset of SolU (15, 21). Patients with mild disease had a substantially higher probability (72.1%) of responding positively to nsAH, compared to those with severe disease (3.5%). While nsAH may be suitable for patients with mild disease, modern targeted therapies may be needed for more severe cases. Thus, patients with moderate-to-severe disease may require earlier consideration of second-line therapies, such as omalizumab.

Additionally, our analysis showed that patients diagnosed solely based on clinical symptoms had a markedly lower probability (19.2%) of responding to antihistamines. This could indicate that symptom-based diagnosis may encompass a heterogeneous patient population with varying underlying pathophysiology. A comprehensive diagnostic work-up with photoprovocation testing seems to be beneficial for treatment response, as suggested by our data.

Our study has a few limitations. i) The diagnosis of SolU was self-reported through the questionnaire, and the information given was not confirmed by a physician. Notably, only 38.2% of patients reported having the diagnosis confirmed by photoprovocation. ii) The diagnostic uncertainty observed in some patients may impact the analysis of treatment responses. iii) A selection bias cannot be ruled out, as patient groups, where recruitment was performed, may attract more severely affected patients. iv) Only a few male patients were included; however, this largely reflects the sex distribution of SolU, which occurs more frequently in women. Additionally, women may access health-related items on Facebook more often than men. v) Although 63 patients stated that they knew their action spectrum, only 42 underwent light provocation testing, suggesting that the reported action spectrum may be unreliable. vi) No validated patient-reported outcome measures assessing disease severity or quality of life were used. Instead, we chose single Likert scale questions to evaluate these parameters, keep the questionnaire short, and reduce the number of potential dropouts. vii) Although the sample size was large, considering that SolU is a rare disease, our study was underpowered for sub-analysis (particularly for treatments other than antihistamines and omalizumab, and for logistic regression models). viii) The survey questionnaire and its translations have not been validated.

To address these issues, future studies should enroll patients with a confirmed diagnosis of SolU by photoprovocation, including the determination of the triggering wavelengths. These studies should use validated patient-reported outcome measures, such as the Urticaria Control Test, the Dermatology Life Quality Index, or the Skindex-29. A multicentric approach is likely needed to achieve a sufficient sample size for adequately powered analysis.

Overall, SolU is perceived as severe by the majority of patients, leading to a high impairment of health-related quality of life. Diagnostic light provocation is only performed in a limited number of patients. In real-life settings, the therapies performed, including off-label treatments, are not sufficient to reach complete remission of symptoms in most patients. Thus, the development of effective therapies and exploration to further personalize therapeutic strategies for SolU patients is of utmost importance to achieve better care for this highly burdened patient population.

## Data Availability

The raw data supporting the conclusions of this article will be made available by the authors, without undue reservation.

## References

[1] McSweeney SM Kloczko E Chadha M Sarkany R Fassihi H Tziotzios C. Systematic review of the clinical characteristics and natural history of solar urticaria. J Am Acad Dermatol. (2023) 89:138–40. doi: 10.1016/j.jaad.2023.01.039, PMID: 36796725

[2] Munoz M Kiefer LA Pereira MP Bizjak M Maurer M. New insights into chronic inducible urticaria. Curr Allergy Asthma Rep. (2024) 24:457–69. doi: 10.1007/s11882-024-01160-y, PMID: 39028396 PMC11297124

[3] Hamel R Mohammad TF Chahine A Joselow A Vick G Radosta S. Comparison of racial distribution of photodermatoses in USA academic dermatology clinics: A multicenter retrospective analysis of 1080 patients over a 10-year period. Photodermatol Photoimmunol Photomed. (2020) 36:233–40. doi: 10.1111/phpp.12543, PMID: 32104953

[4] Haylett AK Koumaki D Rhodes LE. Solar urticaria in 145 patients: Assessment of action spectra and impact on quality of life in adults and children. Photodermatol Photoimmunol Photomed. (2018) 34:262–8. doi: 10.1111/phpp.12385, PMID: 29533487

[5] Rizwan M Reddick CL Bundy C Unsworth R Richards HL Rhodes LE. Photodermatoses: environmentally induced conditions with high psychological impact. Photochem Photobiol Sci. (2013) 12:182–9. doi: 10.1039/c2pp25177a, PMID: 22961505

[6] Jong CT Finlay AY Pearse AD Kerr AC Ferguson J Benton EC. The quality of life of 790 patients with photodermatoses. Br J Dermatol. (2008) 159:192–7. doi: 10.1111/j.1365-2133.2008.08581.x, PMID: 18460025

[7] Zuberbier T Abdul Latiff AH Abuzakouk M Aquilina S Asero R Baker D. The international EAACI/GA(2)LEN/EuroGuiDerm/APAAACI guideline for the definition, classification, diagnosis, and management of urticaria. Allergy. (2022) 77:734–66. doi: 10.1111/all.15090, PMID: 34536239

[8] Snast I Lapidoth M Uvaidov V Enk CD Mazor S Hodak E. Real-life experience in the treatment of solar urticaria: retrospective cohort study. Clin Exp Dermatol. (2019) 44:e164–e70. doi: 10.1111/ced.13960, PMID: 30828851

[9] Maurer M Zuberbier T Metz M. The classification, pathogenesis, diagnostic workup, and management of urticaria: an update. Handb Exp Pharmacol. (2022) 268:117–33. doi: 10.1007/164_2021_506, PMID: 34247278

[10] Parkin D Ling TC Ayer J Rhodes LE Rutter KJ. ‘Omalizumab changed my life’: a patient perspective on solar urticaria. Clin Exp Dermatol. (2024) 49:935–6. doi: 10.1093/ced/llae074, PMID: 38491908

[11] Griffin LL Haylett AK Rhodes LE. Evaluating patient responses to omalizumab in solar urticaria. Photodermatol Photoimmunol Photomed. (2019) 35:57–65. doi: 10.1111/phpp.12434, PMID: 30338865

[12] Maurer M Metz M Brehler R Hillen U Jakob T Mahler V. Omalizumab treatment in patients with chronic inducible urticaria: A systematic review of published evidence. J Allergy Clin Immunol. (2018) 141:638–49. doi: 10.1016/j.jaci.2017.06.032, PMID: 28751232

[13] Soegiharto R Alizadeh Aghdam M Sorensen JA van Lindonk E Bulut Demir F Mohammad Porras N. Omalizumab is effective and safe in chronic inducible urticaria (CIndU): Real-world data from a large multi-national UCARE study. Allergy. (2025) 80:489–99. doi: 10.1111/all.16334, PMID: 39377745 PMC11804303

[14] Exposito-Serrano V Curto-Barredo L Aguilera Peiro P Gomez Armayones S Serra-Baldrich E Spertino J. Omalizumab for the treatment of chronic inducible urticaria in 80 patients. Br J Dermatol. (2021) 184:167–8. doi: 10.1111/bjd.19425, PMID: 32730636

[15] Pesque D Ciudad A Andrades E Soto D Gimeno R Pujol RM. Solar urticaria: an ambispective study in a long-term follow-up cohort with emphasis on therapeutic predictors and outcomes. Acta Derm Venereol. (2024) 104:adv25576. doi: 10.2340/actadv.v104.25576, PMID: 38189220 PMC10789168

[16] Aubin F Avenel-Audran M Jeanmougin M Adamski H Peyron JL Marguery MC. Omalizumab in patients with severe and refractory solar urticaria: A phase II multicentric study. J Am Acad Dermatol. (2016) 74:574–5. doi: 10.1016/j.jaad.2015.11.021, PMID: 26892659

[17] Lyons AB Peacock A Zubair R Hamzavi IH Lim HW. Successful treatment of solar urticaria with UVA1 hardening in three patients. Photodermatol Photoimmunol Photomed. (2019) 35:193–5. doi: 10.1111/phpp.12447, PMID: 30576021

[18] Haylett AK Nie Z Brownrigg M Taylor R Rhodes LE. Systemic photoprotection in solar urticaria with alpha-melanocyte-stimulating hormone analogue [Nle4-D-Phe7]-alpha-MSH. Br J Dermatol. (2011) 164:407–14. doi: 10.1111/j.1365-2133.2010.10104.x, PMID: 20969564

[19] Aubin F Porcher R Jeanmougin M Leonard F Bedane C Moreau A. Severe and refractory solar urticaria treated with intravenous immunoglobulins: a phase II multicenter study. J Am Acad Dermatol. (2014) 71:948–53.e1. doi: 10.1016/j.jaad.2014.07.023, PMID: 25135650

[20] Caccialanza M Recalcati S Piccinno R. Oral polypodium leucotomos extract photoprotective activity in 57 patients with idiopathic photodermatoses. G Ital Dermatol Venereol. (2011) 146:85–7., PMID: 21505393

[21] McSweeney SM Sarkany R Fassihi H Tziotzios C McGrath JA. Pathogenesis of solar urticaria: Classic perspectives and emerging concepts. Exp Dermatol. (2022) 31:586–93. doi: 10.1111/exd.14493, PMID: 34726314

