# Supplementary material for: Current treatment practices and efficacy in solar urticaria: insights from a patient survey
Source: Front Immunol. 2025 Nov 4;16:1683524. doi: 10.3389/fimmu.2025.1683524 (PMC12623372; doi:10.3389/fimmu.2025.1683524)
Supplement: Supplementary file 1 [file Table1.docx]

**Therapies and their outcomes in patients with Solar Urticaria: The patient perspective**

1. **How were you diagnosed with solar urticaria? (Please choose one option)**

- by a physician based on light-provocation-testing
- by a physician based on clinical symptoms
- I or my physician suspect solar urticaria but it has not been formally diagnosed yet
- I do not have solar urticaria

[If patient chooses “I do not have solar urticaria” further questions will not be displayed]

1. **Do you know to what type of light you react to? (Please choose one option)**

- No
- Yes

[If patient chooses “Yes” patients will be asked to choose the possible light options that apply within question 3. If patient chooses “No” question 3 will not be displayed].

1. **You know the type of light you react to; please choose all types of light you are reacting to. (Please choose all that apply)**

- Visible light
- Ultraviolet-A
- Ultraviolet-B
- Infrared light
- Other: _____

1. **What is your gender? (Please choose one option)**

- female
- Male
- Other

1. **How old are you (in years):** ________

1. **When did you first notice the symptoms of your Solar Urticaria? (Month/Year)?** ______/ ______
2. **When were you first diagnosed with Solar Urticaria? (Month/Year)?** _/_______
3. **How long can you be exposed to (sun)light before you start to react?**

- less than 1 minute
- 1 to 5 minutes
- 6 to 10 minutes
- 11 to 20 minutes
- More than 20 minutes

1. **Overall, how much has your Solar Urticaria affected your quality of life?**

- extremely strong
- very strong
- moderate
- little
- not at all

1. **Overall, how severe is your Solar Urticaria?**

- extremely severe
- severe
- moderate
- mild
- extremely mild

1. **Have you ever treated your Solar Urticaria?**

- No
- Yes

[If patient chooses „Yes“ the following questions 12 to 36 will be shown depending on the selected answers]

1. **Have you been taking second generation antihistamines/ non-sedating antihistamines (e.g. Loratadin®, Cetirizine®, Ebastin®, Telfast®, Aerius®, Urtimed®)? (Please choose one option)**

- No
- Yes

1. **How did you take your second generation antihistamines? (please choose all that apply)**

- on demand
- once daily
- more than once daily

[If patient responded „on demand“ question 14 will be shown]

[If patient responded „once daily“ question 15 will be shown]

[If patient responded „more than once daily“ question 16 will be shown]

1. **How do you rate your response to the treatment with „on demand“ second generation antihistamines?**

- symptoms worsened
- no change
- slight improvement
- moderate improvement
- significant improvement
- complete control

1. **How do you rate your response to the treatment with “once daily intake” of second generation antihistamines?**

- symptoms worsened
- no change
- slight improvement
- moderate improvement
- significant improvement
- complete control

1. **How do you rate your response to the treatment with “more than once” daily second generation antihistamines?**

- symptoms worsened
- no change
- slight improvement
- moderate improvement
- significant improvement
- complete control

1. **Have you ever taken first generation antihistamines/ sedating antihistamines (e.g. Clemastin, Dimetinden)?**

- No
- Yes

1. **How did you take your first generation antihistamines? (please choose all that apply)**

- on demand
- once daily
- more than once daily

[If patient responded „on demand“ question 19 will be shown]

[If patient responded „once daily“ question 20 will be shown]

[If patient responded „more than once daily question 21 will be shown]

1. **How do you rate your response to the treatment with „on demand“ second generation antihistamines?**

- symptoms worsened
- no change
- slight improvement
- moderate improvement
- significant improvement
- complete control

1. **How do you rate your response to the treatment with “once daily intake” of second generation antihistamines?**

- symptoms worsened
- no change
- slight improvement
- moderate improvement
- significant improvement
- complete control

1. **How do you rate your response to the treatment with “more than once” daily second generation antihistamines?**

- symptoms worsened
- no change
- slight improvement
- moderate improvement
- significant improvement
- complete control

1. **Have you ever taken oral cortisone (e.g. Decortin) to treat your Solar Urticaria?**

- No
- Yes

[If patient chooses “YES” the following question 23 will be shown]

1. **How do you rate your response to the treatment with oral cortisone?**

- symptoms worsened
- no change
- slight improvement
- moderate improvement
- significant improvement
- complete control

1. **Have you ever taken Cyclosporine (e.g. Immunosporin®, Cicloral^®^) to treat your Solar Urticaria?**

- No
- Yes

[If patient chooses “YES” the following question 25 will be shown]

1. **How do you rate your response to the treatment with Cyclosporine?**

- symptoms worsened
- no change
- slight improvement
- moderate improvement
- significant improvement
- complete control

1. **Have you ever received Omalizumab** **(Xolair®) to treat your Solar Urticaria?**
   - - No
     - Yes

[If patient chooses “YES” the following question 29 will be shown]

1. **What doses have you received of Omalizumab (Xolair®) monthly? (please choose all that apply)**

- I don’t know
- less than 300 mg (1 injection)
- 300 mg (2 injections)
- more than 300 mg (more than 2 injections)

[If patient responded „I don’t know“ question 28 will be shown]

[If patient responded „less than 300 mg“ question 29 will be shown]

[If patient responded „300 mg“ question 30 will be shown]

[If patient responded „more than 300 mg“ question 31 will be shown]

1. **How do you rate your response to the treatment with Omalizumab (Xolair®)?**

- symptoms worsened
- no change
- slight improvement
- moderate improvement
- significant improvement
- complete control

1. **How do you rate your response to the treatment with less than 300 mg monthly Omalizumab (Xolair®)?**

- symptoms worsened
- no change
- slight improvement
- moderate improvement
- significant improvement
- complete control

1. **How do you rate your response to the treatment with 300 mg monthly Omalizumab (Xolair®)?**

- symptoms worsened
- no change
- slight improvement
- moderate improvement
- significant improvement
- complete control

1. **How do you rate your response to the treatment with more than 300 mg monthly Omalizumab (Xolair®)?**

- symptoms worsened
- no change
- slight improvement
- moderate improvement
- significant improvement
- complete control

1. **Have you received UVB/PUVA “Hardening” Phototherapy for your Solar Urticaria?**
   - - No
     - Yes

[If patient chooses “YES” the following question 33 will be shown.]

1. **How do your rate your response to the treatment with UVB/PUVA “Hardening” Phototherapy?**

- symptoms worsened
- no change
- slight improvement
- moderate improvement
- significant improvement
- complete control

1. **Have you taken oral Polypodium leucotomes to treat your Solar Urticaria?**

- No
- Yes

[If patient chooses “YES” the following question 37 will be shown.]

1. **How do you rate your response to the treatment with Polypodium leucotomes?**

- symptoms worsened
- no change
- slight improvement
- moderate improvement
- significant improvement
- complete control

**In the following you will have the possibilty to add treatment you received that have not been mentioned her. Per question you have the possibility to provide information on the name and dose. As many questions will be displayed as additional medication you have taken until you choose answer „not more treatments to add“**

1. **How do you rate the response of ____________________________________________________________________________________________________________________________________________________________________________________________________(please provide name and dose) as a treatment?**

- symptoms worsened
- no change
- slight improvement
- moderate improvement
- significant improvement
- complete control

**After completing to question 36 the following options will be displayed.**

- more medication/treatment to add
- not more treatments to add

[If patient chooses “more medication/treatment to add” the following question 36 and after completing the the options “more medication/treatment to add” and “finish questionnaire” will be shown. This will be repeated until the patient chooses the option “ not mor treatments to add”. Then the following words will be shown]

**Thank you for participating and spending your time on this online survey about Solar Urticaria!**
